# Privacy-Preserving Social Ambiance Measure From Free-Living Speech Associates With Chronic Depressive and Psychotic Disorders

**DOI:** 10.3389/fpsyt.2021.670020

**Published:** 2021-08-11

**Authors:** Wenwan Chen, Ashutosh Sabharwal, Erica Taylor, Ankit B. Patel, Nidal Moukaddam

**Affiliations:** ^1^Department of Electrical and Computer Engineering, Rice University, Houston, TX, United States; ^2^Menninger Department of Psychiatry, Baylor College of Medicine, Houston, TX, United States; ^3^Department of Neuroscience, Baylor College of Medicine, Houston, TX, United States

**Keywords:** social isolation, social ambiance, mental disorders, objective measures, speech, wearable sensors

## Abstract

A social interaction consists of contributions by the individual, the environment and the interaction between the two. Ideally, to enable effective assessment and interventions for social isolation, an issue inherent to depressive and psychotic illnesses, the isolation must be identified in real-time and at an individual level. However, research addressing sociability deficits is largely focused on determining loneliness, rather than isolation, and lacks focus on the richness of the social environment the individual revolves in. In this paper, We describe the development of an automated, objective and privacy-preserving Social Ambiance Measure (SAM) that converts unconstrained audio recordings collected from wrist-worn audio-bands into four levels, ranging from none to active. The ambiance levels are based on the number of simultaneous speakers, which is a proxy for overall social activity in the environment. Results show that social ambiance patterns and time spent at each ambiance level differed between participants with depressive or psychotic disorders and healthy controls. Individuals with depression/psychosis spent less time in diverse environments and less time in moderate/active ambiance levels. Moreover, social ambiance patterns are found associated with the severity of self-reported depression, anxiety symptoms and personality traits. The results in this paper suggest that objectively measured social ambiance can be used as a marker of sociability, and holds potential to be leveraged to better understand social isolation and develop effective interventions for sociability challenges, thus improving mental health outcomes.

## 1. Introduction

It is now well-appreciated that, along with biological and psychological factors, social factors contribute to negative mental health outcomes (1). Social isolation is predictive of greater mental health difficulties for both elders (2) and children (3). Socially isolated individuals are more likely to suffer from depression, loneliness, stress and anxiety (4). Deficits in sociability, and specific elements of social interaction and role functioning, are essential components of mental illness, though the mechanisms of developing sociability impairments may be different across disorders (such as depressive disorders, psychotic disorders, autism spectrum, attention-deficit disorders, etc.).

A social interaction consists of elements brought by the individual, the environment, and the interaction between the two. While the first element focuses on developing and maintaining relationships (e.g., the Global Functioning Scale, the First Episode Social Functioning Scale) (5), the second addresses the environment and ambiance in which the social interactions of interest are happening. Loosely defined as “the character and atmosphere of a place,” ambiance describes the atmosphere created by nearby people and may reflect the inclination for companionship. Generally, socially isolated individuals spend less time around people so the tendency of becoming isolated can be captured by the social ambiance changes. Higher cohesion in neighborhoods (6) are associated with less loneliness, less isolation, and improved sociability (7). Moreover, research shows that a richer social ambiance is associated with better mental health (8), and enriching social ambiance is a fundamental element in the treatment of mental illness (9), whether by social skills training or cognitive behavioral therapy (10). The mere presence of another individual can alleviate stress, but if a person is uncomfortable around others, lacks the ability to initiate/maintain a conversation, or to initiate social activity, this refuge will be absent from their lives (11).

The development of wearable sensors facilitates the objective measurement of social ambiance. Different from subjective measures that are prone to bias and recall mistakes, sensor-based methods enable long-term observation without putting extra burdens on participants. Researchers in (12) leverage the phone's microphone to measure local business ambiance by inferring the occupancy and human chatter levels, the music type, as well as the music and noise levels in the business. The CrossCheck study (13) investigates the relationship between passive smartphone sensor data and mental health changes. Ambient volume was utilized to represent the context of the participant's acoustic environment, and was found to be associated with Ecological Momentary Assessment (EMA) scores. In (14), the authors measured ambiance by calculating the number and duration of conversation students were around in these spaces. Their results showed that higher depression scores were associated with fewer conversations.

Despite the potential opportunities provided by wearable sensors, the measurement of social ambiance has been challenging for three reasons. First, most existing methods fail to capture transient social ambiance patterns since they only provide coarse-scale and aggregated information. Second, fine-scale methods rely on speech analysis by human researchers and hence cannot be implemented in clinical context, e.g., due to a combination of privacy constraints and high human effort. Third, to develop automated methods for unconstrained analysis, abundant *labeled* data is required to train artificial intelligence algorithms. Currently there are no such datasets available that capture the diverse audio environments encountered during the day.

**Figure 1:**
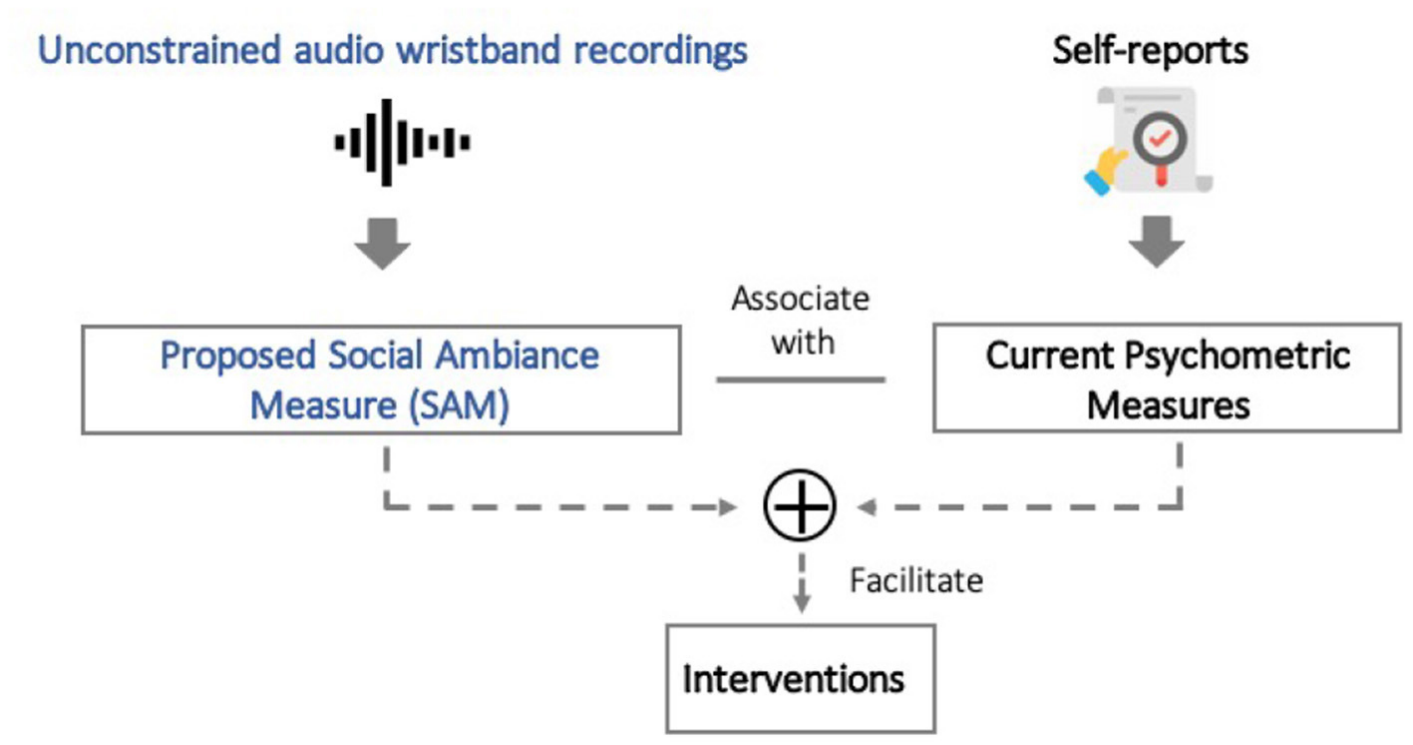
In this paper, we study if social ambiance measure (SAM) associate with psychometrics measures. The new findings could empower new in-time interventions for improving mental health outcomes.

To address the above challenges, in this manuscript, we establish the feasibility of measuring social ambiance objectively, and test the hypothesis that objectively measured social ambiance can be used as a marker of sociability. Specifically, we propose a privacy-preserving social ambiance measure (SAM), derived from wearable sensors that collect unconstrained audio recordings. We use the number of concurrent speakers as a proxy for social ambiance since speech overlaps are prevalent in most social scenarios and create a type of sound texture that represents the atmosphere created by people nearby. To evaluate relationship between social ambiance patterns and mental health, we conducted a pilot study (Figure 1) to compare individuals with chronic depression, chronic psychotic disorders vs. healthy controls. The proposed SAM converts unconstrained audio recordings into four levels—quiet (no speech), low, moderate, and high, thereby using the number of concurrent speakers as proxy for social ambiance—definitions of speaker numbers are listed in the section 2. The conversion of audio band data into the four ambiance levels is performed at 5 s intervals, thereby achieving a high-resolution measure of changes in ambiance throughout the day. These short duration measurement can then be aggregated in diverse ways; in this paper, we study the fraction of time spent in each level during the course of the week-long pilot study as described below.

The proposed method captures fine-grained deep learning based algorithm directly maps speech to reconstitute ambiance information into the four pre-set levels without *any content analysis* to ensure participant privacy. To ensure high accuracy in converting unconstrained audio to the proposed measure, we optimized deep neural network based algorithms on open source datasets that we synthesized to mimic daily environments like home, workplace and outdoors. During the whole process, no content of participant recordings was analyzed or listened to either by a human or an algorithm.

## 2. Methods

### 2.1. Participants

For our pilot study, a total of 32 participants were recruited that included 11 outpatients with major depressive disorder (no psychotic features), 8 outpatients with schizophrenia or schizoaffective disorders and 13 age-matched controls. Participant demographics are presented in Table 1.

**Table 1:** Participants characteristics.

		**Control group** **(** ***N*** **= 13)**	**Depression group** **(** ***N*** **= 11)**	**Psychosis group** **(** ***N*** **= 8)**
Age		45.3 (11.2)	52.4 (13.0)	35.3 (10.5)
Gender	Female	8 (61.5%)	8 (72.7%)	3 (37.5%)
	Male	5 (38.5%)	3 (27.3%)	5 (62.5%)
Race	Black or African American	6 (46.2%)	6 (54.5%)	4 (50.0%)
	Hispanic	4 (30.8%)	1 (9.1%)	2 (25.0%)
	White	1 (7.7%)	3 (27.3%)	2 (25.0%)
	Asian	1 (7.7%)	0 (0.0%)	0 (0.0%)
	Indian	1 (7.7%)	0 (0.0%)	0 (0.0%)
	Iranian	0 (0.0%)	1 (9.1%)	0 (0.0%)
Employment status	Employed	13 (100.0%)	4 (36.4%)	0 (0.0%)
	Unemployed	0 (0.0%)	7 (63.6%)	8 (100.0%)
Marital status	Single	5 (38.5%)	4 (36.4%)	7 (87.5%)
	Married	8 (61.5%)	2 (18.2%)	0 (0.0%)
	Separated	0 (0.0%)	1 (9.1%)	0 (0.0%)
	Divorced	0 (0.0%)	3 (27.3%)	1 (12.5%)
	Widowed	0 (0.0%)	1 (9.1%)	0 (0.0%)
Education	High school	1 (7.7%)	5 (45.5%)	8 (100.0%)
	Associate degree	3 (23.1%)	1 (9.1%)	0 (0.0%)
	Bachelor's degree	6 (46.2%)	4 (36.4%)	0 (0.0%)
	Graduate school	3 (23.1%)	1 (9.1%)	0 (0.0%)

### 2.2. Procedures

The study was approved by the Institutional Review Boards (IRB) for Baylor College of Medicine, Harris Health System and Rice University. Intake procedures included ascertaining diagnoses by obtaining medical records for participants in the depression and psychosis groups. Participants had to be stable for outpatient management and not to have had hospitalizations within a year. All participants in the non-control groups were stable on medication regimens. Depression and anxiety were assessed with the Patient Health Questionnaire-9 (PHQ-9) and Generalized Anxiety Disorder-7 (GAD-7). Physical and Social Network (SPN)/Online Social Network Mapping were drawn manually during the interview as every participant summarized their social network (participants were asked to indicate up to ten people they interact with the most closely and the degree of closeness as well as the frequency of contact). Personality traits were measured with the Mini-IPIP Personality scale (15). Starting in March 2018, the study was 1-week long for each participant. For audio recordings, all participants were instructed to wear their wrist-worn audio-bands between the hours of 8 a.m. to 8 p.m. daily. Each wristband has up to 20 h of battery life and can store audio recordings of up to 90 h. Therefore, the wristbands needed to be charged daily. The average charging time is 1 h from empty to full. Participants were also asked to download the HeathSense app developed and deployed in our previous research (16). Phone call logs and text logs were collected through the app to capture social interactions via phone. For the purpose of this paper, phone-based interactions are considered as remote interaction contrasting them with in-person interaction.

### 2.3. Measures

#### 2.3.1. Social Ambiance Measure (SAM)

To mimic human perception of social ambiance, several factors should be considered. First, humans experience the ambiance of a place, often without actually counting the number of nearby people. Second, humans are more discriminative when there are fewer people around. That is, we tend to and can estimate the size of small groups much better than large groups. Thus, we classified detected speech into different levels—quiet, low, moderate, and high social ambiance levels. Since one aspect of social ambiance can be measured by the number of socializing people in the environment, we used the number of concurrent speakers as a proxy for social ambiance and extracted ambiance patterns objectively from audio-recordings. No content analysis was performed to preserve participants' privacy. Following above principles, we defined the social ambiance measure (SAM) as a four-dimensional vector with following four ambiance levels:

*Ambiance Level 0-None* (AL-0): From the recorded audio data, the fraction of time (measured in % of total time) no human speech was detected. AL-0 measures the fraction participant was not around people.

*Ambiance Level 1-Low* (AL-1): From the recorded audio data, the fraction of time (measured in % of total time) only 1 speaker is detected. AL-1 could arise from either participant talking to themselves or on the phone or with one person talking close to them (e.g., on the phone).

*Ambiance Level 2-Moderate* (AL-2): From the recorded audio data, the fraction of time (measured in % of total time) 2–5 speakers are detected. AL-2 represents that the participant was around a medium size group.

*Ambiance Level 3-High* (AL-3): From the recorded audio data, the fraction of time (measured in % of total time) more than 5 speakers detected. AL-3 indicates that the participant was around a large size group.

In addition, we defined a derived measure called entropy based on information theory (17), to measure the variability of the time the participant spent at different ambiance levels. Entropy was calculated as:

(1) $$\begin{array}{l} \text{Entropy}=-\underset{i}{\sum }p_{i}\cdot \log p_{i} \end{array}$$

where *p*_*i*_ represents the probability of Ambiance Level *i*, computed as $p_{i}=\frac{\text{AL-}i}{100}$. Higher entropy indicated that the participant spent time more uniformly across different ambiance levels, while lower entropy indicated greater inequality in the time spent across different ambiance levels.

The four dimensions (a) AL-0, (b) AL-1, (c) AL-2, (d) AL-3, and the derived measure (e) entropy, were averaged across a week for each participant. Note that human voices from televisions or radios were not excluded since the research staff was not allowed to hear the recording (due to privacy restrictions) and there is no easy way to algorithmically distinguish between TV/radio voices and in-person voices.

#### 2.3.2. Psychometric and Personality Measures

For all participants, at the beginning of the study, the Patient Health Questionnaire (PHQ-9) (18) was used to calculate depression severity. Anxiety levels were evaluated with General Anxiety Disorder-7 (GAD-7) (19). In addition, personality factors such as increased neuroticism or decreased extraversion also play a role in decreased social interaction regardless of clinical symptom severity (20). So personality traits (agreeableness, extraversion, neuroticism, openness, consciousness) were measured with the Mini-IPIP Personality scale (15). Note that most patients we recruited rate themselves as severely depressed or anxious. Participants from the depression group had an average PHQ-9 of 19.70 (standard deviation 6.73) and an average GAD-7 of 14.90 (standard deviation 4.75). Participants from the psychosis group had an average PHQ-9 of 15.17 (standard deviation 8.80) and an average GAD-7 of 16.33 (standard deviation 7.84). Moreover, compared with healthy participants, participants diagnosed with mental disorders scored higher on neuroticism and conscientiousness personality traits.

#### 2.3.3. Self-Reported Social Network

At the beginning of the study, participants were asked to list up to ten social contacts, the degree of closeness and frequency of contact. An ego-centric social network can be built from above information, which captures the interactions between the target person and his/her contacts. Such method has been used to visualize social networks (21) and quantify social support (22).

### 2.4. Data Preprocessing

#### 2.4.1. Computing Social Ambiance Patterns

**Figure 2:**
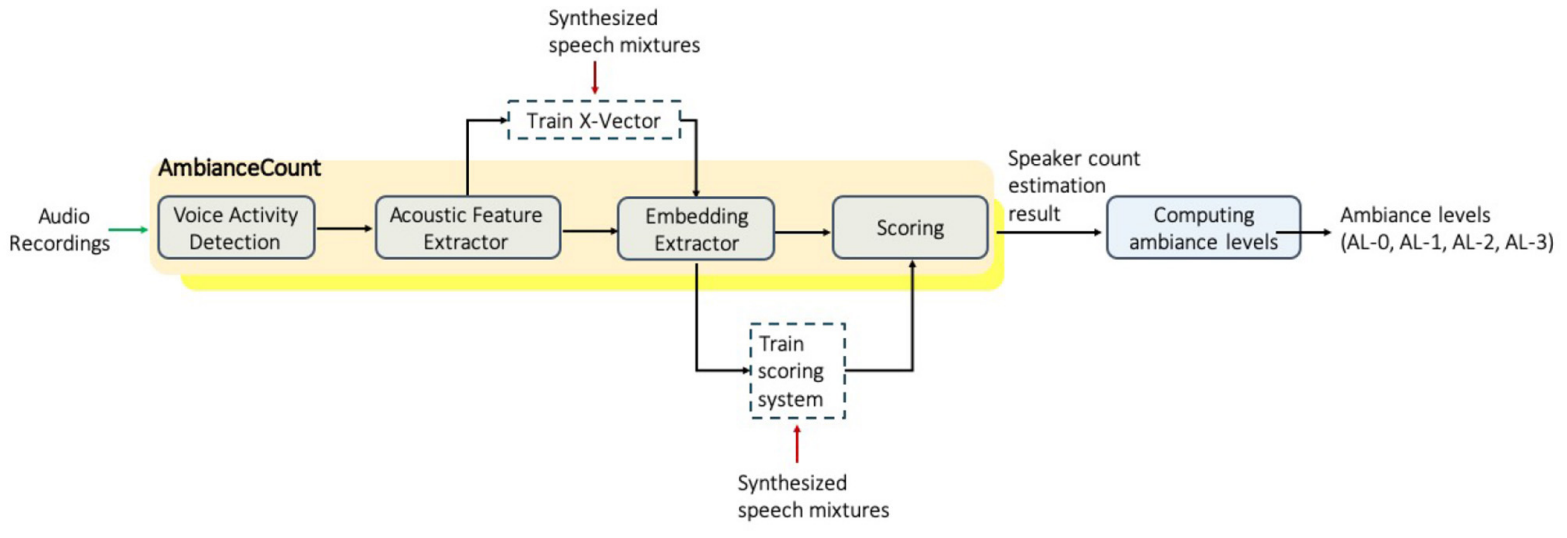
The process of extracting social ambiance patterns from unconstrained audio wristband recordings.

A total of 1,550 h (520 GB) of audio data were collected to extract social ambiance patterns. For privacy concerns, no speech content was listened to or analyzed. Figure 2 summarizes how the raw audio data was processed using a deep-learning-based automated computer algorithm into social ambiance levels, AL-0 to AL-3.

First we applied a voice activity detection algorithm (23) to assess when human speech was nearby. Then the number of concurrent speakers was estimated based on our previous work (24). Finally, speaker count results were mapped to ambiance levels. One notable advantage of our method is that we leveraged public datasets for model development so no content of the clinical dataset was listened to or analyzed, so the procedure was privacy-preserving. This was achieved by training the algorithm on public speech datasets and then apply the developed algorithm to our target clinical data using deep learning techniques.

Controlling for confounding factors: In real-world scenarios, differences in speech patterns and the diversity of background noises can be a confounding factor that reduce the accuracy of concurrent speaker count. To control for above confounding factors and build a robust synthesized dataset for model development, we randomly added noise, reverberation and adjusted speech patterns to simulate real-world scenarios (see Supplementary Section 2.2).

**Figure 3:**
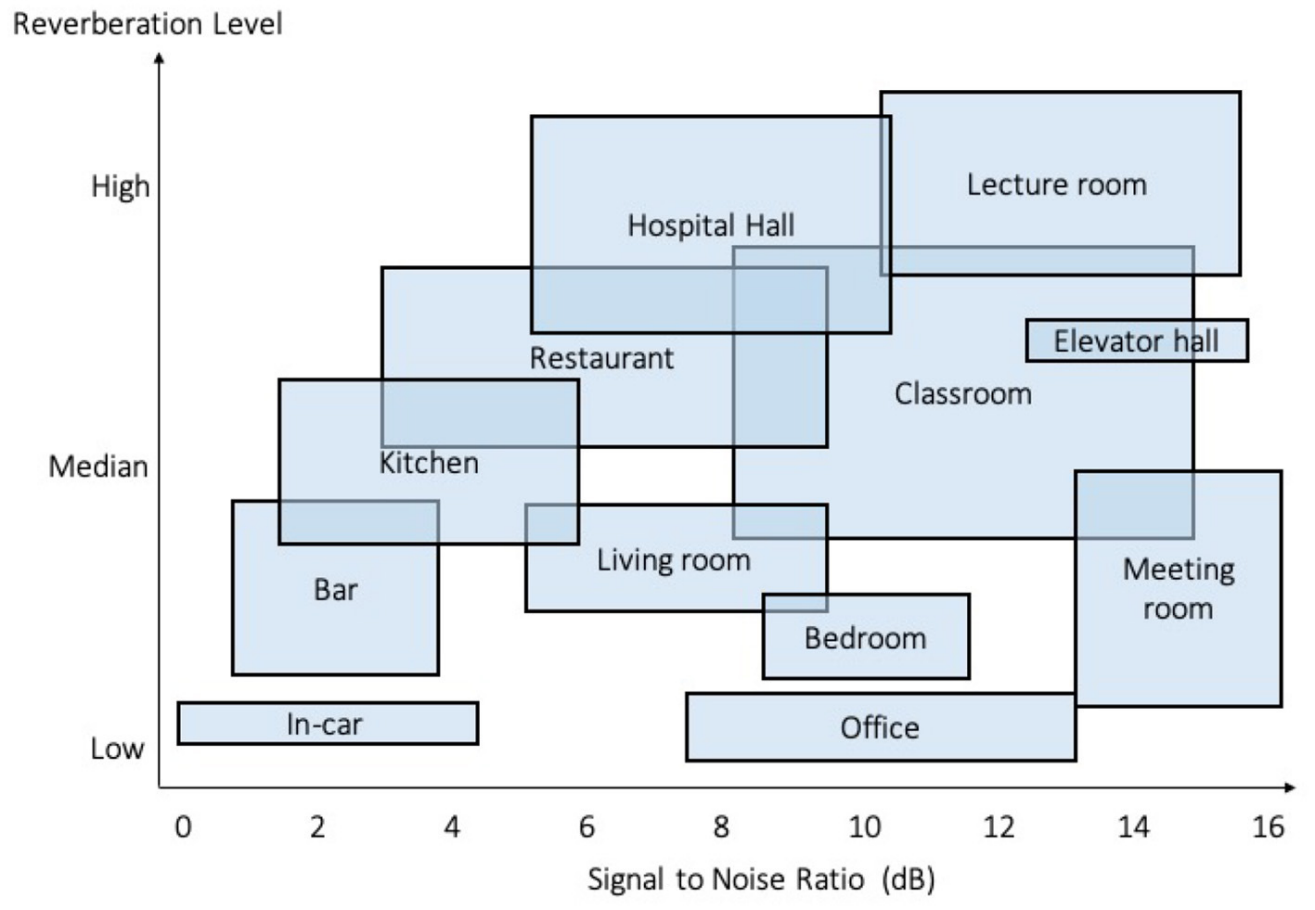
Simulated scenarios in our synthetic datasets, based on the comprehensive analyses of daily acoustic scenarios in terms of noise and reverberation level (27, 28).

Specifically, to simulate various speech patterns where people speak in different volumes and speaking rates, we applied random a volume factor between −3 to +3 dB and a speaking rate factor from −0.9 to + 0.8 dB to synthesized speech. To cover different acoustic scenarios, MUSAN (25) dataset was leveraged to simulate background and foreground noises, including sound of things (e.g., dialtones, fax machine noises), natural sounds (e.g., thunder, wind), and music without vocal(e.g., Western art music and popular genres). Finally, the speech mixtures were reverberated using RIRs (26) dataset to simulate different room settings (e.g., small room, medium room, and large room). Based on the studies (27, 28) that conducted comprehensive analyses of daily acoustic scenarios in terms of noise and reverberation level, our synthetic datasets were able to simulate various scenarios like bedroom, kitchen, meeting room, office, classroom, restaurant, hospital hall, etc., as shown in Figure 3.

##### 2.4.1.1. Acoustic Feature Extractor

To capture significant sound characteristics and differentiate between speech mixtures, acoustic features were extracted from recordings using Kaldi toolkit (29). We combined two types of features, Filter Banks and Pitch, which mimic the non-linear human perception of sound and captures its fundamental frequency, respectively.

##### 2.4.1.2. Embedding Extractor

Acoustic features were then fed into a deep neural network to extract embeddings that can best discriminate speech mixtures. The embedding extractor was based on the X-vector architecture (30), developed using Kaldi (29) for acoustic feature extraction and PyTorch (31) for building neural networks (see Supplementary Section 1). According to the IRB consent form, no content information would be listened to or analyzed. Therefore, the embedding extractor was trained and validated on datasets synthesized from open speech corpus LibriSpeech (32) (see Supplementary Section 2.1).

##### 2.4.1.3. Scoring

A backend scoring system was developed to output the number of concurrent speakers by comparing distances between embeddings (see Supplementary Section 1.3). Also, trained on the above synthetic datasets and, it helped the algorithm generalize on new data. Experiments in (24) showed that the algorithm was able to generalize well on unseen data with different speakers, speech content, and even languages.

##### 2.4.1.4. Computation of Ambiance Levels

For each participant, the duration of each ambiance level was aggregated on a daily basis. Then, the frequency of each ambiance level was calculated per day and averaged across a week, which quantified the ambiance information for a participant.

##### 2.4.1.5. Entropy of Ambiance Levels

Given the daily frequency of ambiance levels for a specific participant, entropy was calculated to capture the diversity of the environments during the day. Higher entropy represented a more diverse environment.

##### 2.4.1.6. Performance

The accuracy was determined by both voice activity detection (VAD) (23) and concurrent speaker count estimation (24). Apart from LibriSpeech (32), we synthesized two additional datasets from TIMIT (English) (33) and THCHS (Mandarin) (34) to evaluate the performance in uncontrolled environments where noise, new speakers and different languages might degrade the performance. The preparation of synthetic evaluation data follows the procedures mentioned in embedding extractor (see Supplementary Section 2). Table 2 shows the sensitivity and specificity of different synthetic datasets. While the model was trained on LibriSpeech, the performance dropped only slightly on two additional datasets, which indicates that the model was robust to environmental noise, and generalized well on unseen data.

**Table 2:** Performance dropped only slightly on two additional synthetic datasets TIMIT (English) and THCHS-30 (Mandarin).

**Source datasets**	**Sound effects**	**Sensitivity (%)**	**Specificity (%)**
LibriSpeech	Noise (25) + Reverberation (26)	82.12	79.35
TIMIT	Noise (25) + Reverberation (26)	81.06	79.27
THCHS-30	Noise (25) + Reverberation (26)	81.42	77.03

*All synthetic data were augmented with noise and reverberation to simulate real-world scenes*.

#### 2.4.2. Mobile Data Processing

Since we aim to quantify the in-person experience, mobile data were leveraged as context information to exclude remote social interactions via phone calls. To protect user privacy, no phone call content was analyzed and phone numbers were encrypted using one-way MD5 hashing. For each user, we calculated the duration of incoming and outgoing phone calls per day.

### 2.5. Statistical Analyses

Phone-call conversations were also recorded by the wristbands. According to phone call logs captured using our mobile logging app, the duration of phone calls made was 11.8% of the total recordings for the control group, 10.2% for the depression group and 9.1% for the psychosis group. Thus, the majority of data captured from the audio-band recordings represents the in-person social ambiance.

We first conducted one-way analyses of variance test (ANOVA) to assess whether there are ambiance differences between the depression, psychosis and control group. The ANOVA tests the null hypothesis, which assumes that participants from three groups are drawn from populations with the same mean values. The F-statistic and p-values produced from ANOVA indicate the group difference and its significance.

We also performed multiple regression analyses to assess if social ambiance patterns were associated with psychometric scores, personality traits and self-report social networks. We used the generalized linear model (GLM) to extend linear regression by allowing response variables to have error distribution models other than a normal distribution. To address the multiple comparisons problem, we applied the Benjamini-Hochberg procedure (BH) (35) to control the false discovery rate (FDR) in our multiple regression analyses.

## 3. Results

### 3.1. Ambiance Differences Between Groups

**Figure 4:**
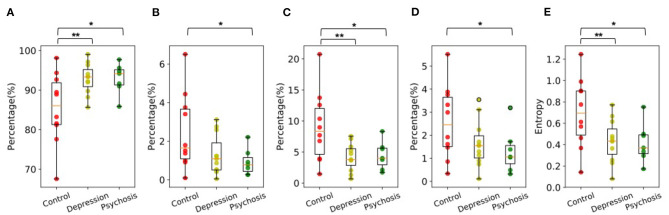
The distribution of **(A)** AL-0, **(B)** AL-1, **(C)** AL-2, **(D)** AL-3, and **(E)** Entropy for control, depression and psychosis group. Group Differences are significant according to one-way ANOVA tests. The brackets show significant results with *p < 0.1, **p < 0.05, and ***p < 0.01.

Figure 4 illustrates that social ambiance patterns extracted from participants with depressive or psychotic disorders were significantly different from healthy controls.

Figure 4A shows that the results of AL-0 for all three groups. The participants from both depression and psychosis groups spent longer duration without any speech around them. According to ANOVA results, compared to the control participants, the difference was significant for depression group with *F* = 6.02, *p* = 0.024, and for psychosis group with *F* = 4.17, *p* = 0.059.

Figure 4B shows that participants from psychosis group had reduced AL-1 compared to the control group. The difference was significant with *F* = 3.90 and *p* = 0.067.

Figure 4C shows that participants from both depression and psychosis groups had reduced AL-2, indicating they spent less time around *moderate* ambiance levels where 2-5 speakers spoke simultaneously. Compared with healthy controls, the differences were significant for depression group with *F* = 6.87, *p* = 0.017, and for psychosis group with *F* = 3.96, *p* = 0.065.

Figure 4D shows that participants from psychosis group had significantly reduced AL-3 compared to the control group, indicating they spent less time around *high* ambiance levels where more than 5 speakers spoke simultaneously. The difference was significant with *F* = 3.38 and *p* = 0.086.

Figure 4E shows that participants from both depression and psychosis groups had significantly reduced entropy. The living environments of participants with depressive or psychotic disorders appeared to be less diverse than healthy controls. Compared with healthy controls, the differences were significant for depression group with *F* = 4.95, *p* = 0.038 and for psychosis group with *F* = 4.15, *p* = 0.060.

### 3.2. Self-Reported Measures

While social ambiance was able to differentiate groups, individual differences were noticed within each group, which might reflect their clinical status, diverse personality traits and size of their social network.

**Figure 5:**
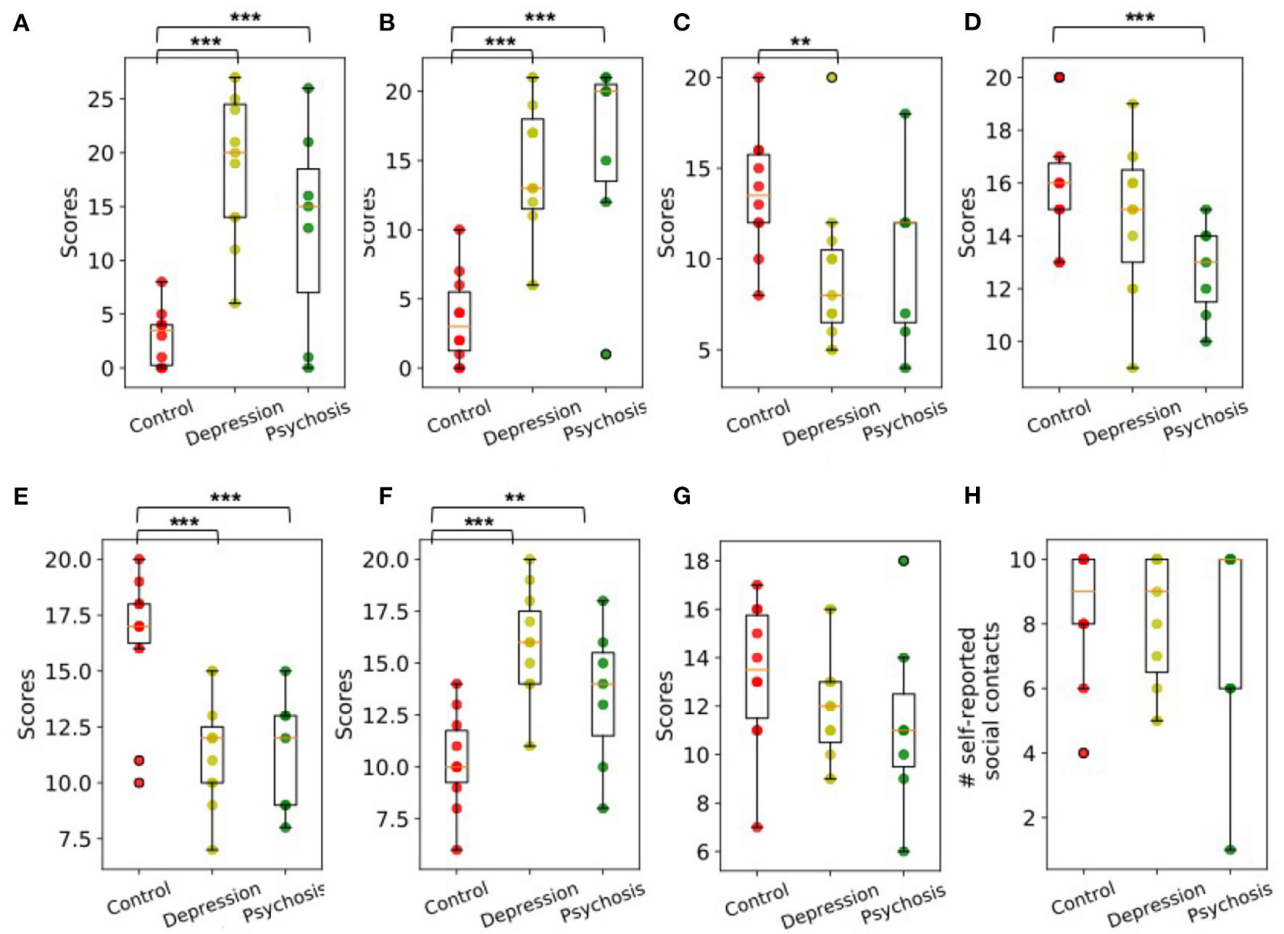
The distribution of **(A)** PHQ-9, **(B)** GAD-7, **(C)** Extraversion, **(D)** Agreeableness, **(E)** Conscientiousness, **(F)** Neuroticism, **(G)** Openness, and **(H)** Number of self-reported social contacts for control, depression, and psychosis group. Group Differences are significant according to one-way ANOVA tests. The brackets show significant results with *p < 0.1, **p < 0.05, and ***p < 0.01.

Figure 5 shows the distribution of the psychometric, personality scores and the number of self-reported social contacts across three groups. Compared with healthy controls, participants from depression group scored higher on PHQ-9 (*F* = 47.25, *p* = 1.472e-06), GAD-7 (*F* = 29.32, *p* = 3.172e-5), neuroticism trait (*F* = 25.74, *p* = 6.748e-5) and lower on personality traits like extraversion (*F* = 6.70 and *p* = 0.018) and conscientiousness (*F* = 15.09, *p* = 9.967e-4). Participants from psychosis group had higher scores on PHQ-9 (*F* = 10.42, *p* = 0.006), GAD-7 (*F* = 21.59, *p* = 3.164e-4), neuroticism trait (*F* = 4.96, *p* = 0.042), and lower scores on agreeableness (*F* = 9.79 and *p* = 0.007) and conscientiousness (*F* = 11.29, *p* = 0.004). No significance difference was observed for the number of self-reported social contacts and openness personality trait across three groups.

### 3.3. Relationship Between Social Ambiance Measure (SAM) and Self-Reported Measures

A generalized linear model (GLM) was used to determine the relationship between social ambiance measure (SAM) and self-reported measures. The most notable finding was that social ambiance patterns, while linked to some personality traits for healthy controls, were found associated with psychometric scores for participants with depressive or psychotic disorders. Table 3 shows that,

**Table 3:** Multiple linear regression analyses of social ambiance measure (SAM) with psychometric measures, personality measures and the number of self-reported social contacts.

		**Psychometric measures**		**mini-IPIP**					
**Ambiance** **patterns**	**Group**	**PHQ-9**	**GAD-7**	**Extraversion**	**Agreeableness**	**Conscientiousness**	**Neuroticism**	**Openness**	**# self-reported** **social contacts**
AL-0	Depression	0.174	0.354	1.114	0.925	1.341	−2.152^*^	1.152	0.405
	Psychosis	−1.622	−3.847^***^	−0.269	0.230	0.116	0.503	−0.708	3.565^***^
	Controll	0.027	1.206	0.325	−0.641	**−2.147**	0.943	−0.678	−0.487
AL-1	Depression	−1.657	−1.337	1.589	1.695^*^	0.872	−1.142	1.831	0.271
	Psychosis	−3.145^***^	−12.800^***^	0.359	0.970	0.632	0.052	0.804	1.774
	Controll	0.026	−0.630	1.642^*^	−0.070	1.457	−0.084	1.861	−0.648
AL-2	Depression	−1.075	−1.822	0.378	1.257	1.027	−3.781^***^	1.712	0.252
	Psychosis	−1.577	−4.051^***^	2.094^**^	0.892	1.012	0.226	1.874	9.131^***^
	Controll	0.184	1.113	−0.617	−0.435	−2.821^**^	0.687	−1.128	0.191
AL-3	Depression	0.821	−0.366	−1.296	−0.922	−0.497	−0.764	−1.900	−0.628
	Psychosis	−1.568	−10.411^***^	−1.365	−0.141	0.294	−0.560	−1.128	5.027^***^
	Controll	−0.481	0.586	−1.174	−0.646	−1.543	0.281	−0.784	0.114
Entropy	Depression	1.245	2.050^**^	1.175	0.315	0.984	−0.428	0.760	0.762
	Psychosis	−0.542	2.462^**^	−0.869	−0.162	−0.468	0.800	−1.423	−0.001
	Controll	0.454	0.549	0.555	0.057	0.945	0.730	−0.104	−1.399

*Figures are Z scores. Bold p < 0.05*,

* *FDR < 0.1*,

** *FDR < 0.05, and*

*** *FDR < 0.01*.

*Depression Group*: (1) entropy was positively associated with GAD-7; (2) AL-1 was positively associated with the agreeableness trait; (3) AL-0 and AL-2 were negatively associated with the neuroticism trait.

*Psychosis Group*: (1) AL-1 was positively associated with the extraversion trait; (2) AL-0, AL-1, AL-2, and AL-3 were negatively associated with GAD-7; (3) entropy was positively associated with GAD-7 (4) AL-2 was positively associated with the extraversion trait. (5) AL-0, AL-2, and AL-3 were positively associated with the number of self-reported social contacts.

*Control Group*: (1) AL-1 was positively associated with the extraversion trait; (2) AL-3 and AL-3 were negatively associated with conscientiousness trait.

## 4. Discussion

In this manuscript, we established the feasibility of measuring social ambiance objectively and unobtrusively, and found social ambiance variability could differentiate between healthy controls with no mental illness and individuals with psychotic or depressive disorders. Results show that the automatically extracted social ambiance patterns were able to differentiate healthy controls from individuals with chronic depressive or psychotic disorders. Compared with the control group, participants from depression and psychosis group spent less time around people and had lower levels of social ambiance, indicating that they were more likely to be socially isolated. Also, participants from depression and psychosis groups were less likely to have diverse environments in which social interactions occurred as well. This is in line with the literature on social cognition in chronic mental illness, but this information was collected via SAM, highlighting the feasibility of objective detection of social isolation, and indicating that objectively measured social ambiance can be used as predictors of mental disorders. These findings can be conceptualized as building blocks and technology validation that can be used in the future for specific mental conditions and mitigation or even prevention of specific sequelae in context of trauma, or mood or psychotic episodes. Of note, while the study of sociability is of intuitive interest to mental illnesses, future studies should also take into account the timing of a sociability “rupture” or derailment, whether it is caused by medical illness, mental illness or other trauma.

Associations between SAM and subjective measures (PHQ-9 and GAD-7) show that the ambiance patterns of participants with depressive or psychotic disorders were linked to the severity of their depression and anxiety symptoms, even though they were all considered suitable for outpatient management and had been in active treatment for at least a year; this is a testimony to the burden of sociability deficits in individuals with chronic disorders that is largely unaddressed despite clinical treatment. We also noticed that for participants from psychosis group, there were positive associations between social ambiance patterns and the number of self-reported social contacts, one possible reason is that participants from the psychosis group had limited living environments compared to other participants so most of their detected ambiance came from their existing social contacts. The above results indicate that objectively measured social ambiance provides a solution for the detection of social isolation with fine granularity, noting that SAM generates 4 numbers (AL-0 to AL-3) every 5 s. This fine resolution information could be leveraged to study finer patterns of social ambiance changes, both at individual and population levels. This will be an important future research direction, as they could be used to enable in-time personalized interventions.

Our study, despite the small sample size, was also able to detect a contribution of personality factors to sociability, which is very promising in terms of assessing individual sociability “sweet spots” (an individual's desired sociability level, matching their comfort level) and tailoring treatments in the future. The effect of personality traits detected was consistent with published literature, and suggesting neuroticism and agreeableness can impact sociability in opposite manners. An individual's disposition can be examined using multiple parameters, including personality and temperament, as well as social cognition frameworks (36) consisting of emotion processing, theory of mind, attributional bias, and social perception. Temperament is the set of neurochemically-defined pre-existing features that dictate how individuals interact with their environment, while personality is thought to be the product of biological and socio-cultural influences (37). For this study, the choice of the personality model for this study was guided by the exploratory nature of the study and small anticipated sample size; a more nuanced model (eg temperament) would not have been conducive to meaningful data analysis at this stage. Future research efforts should take into account temperament measures and highlight the link to SAM.

Different from self-report methods that are prone to bias and recall errors, our method enables long-term and fine-grained observations by continuously capturing the environment with wearable sensors. Abundant information was extracted from passively collected data, with excellent acceptance from participants. Audio recordings collected from wristbands were good data sources since they captured transient social behaviors and kept the detailed information of the acoustic environment. Social ambiance was reconstructed from audio recordings and the privacy of participants was protected since no speech content was listened to or analyzed. Our method can be easily replicated in multiple settings since we do not rely on private clinical data for model development. Deep learning techniques enable the model to be developed on open datasets and transferred to target scenarios. The advantage of objective measurements also lies in avoiding an individual's illness affecting their assessment of their social network size, of the quality of their social interactions, or of their progress in social settings (e.g., depression and lack of belongingness in depressed individuals or paranoia/delusions in individuals with chronic psychosis).

This project is part of a larger attempt to explore the feasibility of ecological momentary interventions based on sociability levels in mental illness: two paradigm shifts are at play in this line of thinking. First, cognitive-behavioral therapies and social skills training are accepted modalities to improve social difficulties individuals with chronic mental illness, but outcome measurement is lacking and not consistent. Second, there is evidence that some social skills training measures (active listening, communicating pleasant or unpleasant emotions, etc.) can be done via an app rather than in-person therapy (10). For optimal in-time interventions, ambiance measurements would be central to the dynamic assessment of intervention results. Group psychotherapy and partial hospitalization programs, as well day programs for chronic psychotic disorders, have long been part of clinical treatment plans, but the explicit goal of measuring social ambiance enrichment, or the contribution of a therapeutic milieu (long a tenet pf psychiatric treatment) have never been formally explored as a treatment measure. Lastly, from a diagnostic perspective, use of SAM or analogous objective measures can conceivably detect pre-morbid symptoms before a first break psychosis or decompensation/start of a depressive episode.

It is necessary to underline that the results are preliminary given the relatively small sample size and 1-week study duration, and there are several limitations in our study. First, the generalizability of the study is limited by the short-term study we conducted. Long-term studies are required to find long-term behavior patterns and predicting clinical outcomes. Second, the relatively small sample size would limit the generalizability for individuals with varying degrees of depression or psychosis. In our study, participants in depression group rated themselves as fairly depressed, and participants with psychosis symptoms had significant residual symptoms. Also, limited by the sample size, participants from the control group are more toward employed and better educated than the depression and psychosis groups. This raises some concern that employment and education level might also play a role in the differences in results between groups. So for future work, we plan to recruit more participants from diverse cultural and educational backgrounds, with matched employment and marital status between groups, and conduct longer-term follow-ups. Additionally, we quantified only one aspect of social ambiance by counting the number of speakers, aiming at establishing the feasibility of measuring social ambiance with wearable sensors. The reason is that such an aspect of social ambiance directly comes from sensory perception, which is reported as a crucial factor in determining Quality of Life and outcomes in clinical practice (38). For future work, we plan to extend the social ambiance measurement by objectively recognizing the emotion, character and atmosphere of people nearby, thus addressing the multi-faceted characteristics of ambiance.

Even with the limited sample, however, glaring differences in levels of social interactions were detected. Over the course of a chronic illness, cumulative absence of social interactions can severely hamper social capital and lifelong relationships. Also, social support is reported to be a mediator between trauma and self-injury behaviors (39), suggesting the importance of social support in coping with lifetime traumatic experiences. Thus, the detection of social isolation deserves close scrutiny as social/functional improvement remains an often un-achieved goal of treatment. Future studies should also examine the difference in ambiance exposures between depressive and psychotic disorders, which likely differ in mechanisms of sociability deficit development, and the impact of effective treatment on these measures. On a larger theoretical scale, the study of sociability as an independent psych-developmental dimension can have implications on how psycho-social functioning in mental illnesses is conceptualized, optimized and managed or treated. It can also have implications on defining normative milestones for sociability by objective measures. As social interactions constitute the building blocks of human interactions, objective normative foundations, against which impairment or deficits can be measured, will be needed. SAM objective measurements, exemplified by this pilot trial, are an essential first step in proving feasibility of building this framework. Sociability dimensions of interest for which objective measurements have to be built/refined include the number of individuals a person interacts with (social network size estimation), speakers in the environment (ambiance levels), and the individual's contribution in a typical conversation. All these parameters are expected to be rooted in an individual's personality, upbringing and interaction styles, and will be impacted by traumatic experiences and mental illness. These objective measurements will then hopefully be studied in the context of the person's subjective perception of said interaction, and related feelings of social anxiety, loneliness or fulfillment.

In conclusion, we verified the feasibility of developing privacy-preserving social ambiance measure (SAM) with chronic depressive and psychotic disorders. The novelty of this study lies in the ability to objectively, privately quantify social ambiance to detect social isolation, and can have far-reaching consequences in understanding and tracking an individual's personalized sociability needs and gaps. Lastly, this approach can have value as it is a non-clinical, non-pharmacological approach that can complement current methods to improve mental health outcomes. As future work, we anticipate that fine-grained analysis of SAM at various illness stages could be used detect behavioral precursors and provide valuable information for early, just-in-time intervention. As shown in Figure 1, SAM, as a non-clinical method, complements psychometric measures by enabling fine-grained and potential long-term follow-up with little burden on patients.

## Data Availability Statement

The original contributions presented in the study are included in the article/Supplementary Material, further inquiries can be directed to the corresponding author/s.

## Ethics Statement

The studies involving human participants were reviewed and approved by Institutional Review Boards (IRB) for Baylor College of Medicine, Harris Health System, and Rice University. The patients/participants provided their written informed consent to participate in this study.

## Author Contributions

WC, AS, and AP analyzed data. WC wrote manuscript. NM and ET implemented the study and ran the data collection. NM and AS designed the study. All authors contributed to manuscript revision, read, and approved the submitted version.

## Conflict of Interest

The authors declare that the research was conducted in the absence of any commercial or financial relationships that could be construed as a potential conflict of interest.

## Publisher's Note

All claims expressed in this article are solely those of the authors and do not necessarily represent those of their affiliated organizations, or those of the publisher, the editors and the reviewers. Any product that may be evaluated in this article, or claim that may be made by its manufacturer, is not guaranteed or endorsed by the publisher.
