# Supplementary material for: Privacy-Preserving Social Ambiance Measure From Free-Living Speech Associates With Chronic Depressive and Psychotic Disorders
Source: Front Psychiatry. 2021 Aug 11;12:670020. doi: 10.3389/fpsyt.2021.670020 (PMC8385275; doi:10.3389/fpsyt.2021.670020)
Supplement: Supplementary file 1 [file Data_Sheet_1.PDF]

Supplementary Material:Privacy-preserving Social Ambiance Measure from Free-living Speech Associate with Chronic Depressive and Psychotic Disorders

In this supplementary material, we describe the deep learning based AmbianceCount algorithm that converts unconstrained audio to four social levels that constitute the proposed social ambiance measure.

1 AMBIANCECOUNT ARCHITECTURE

To capture social ambiance from unconstrained day-long audio, the first step is to extract audio clips where human voices are detected using *Voice Activity Detection* (VAD) (Doukhan et al., 2018). The audio clips without speech are classified as Ambiance Level 0, and Ambiance Levels 1-3 are inferred from the number of concurrent speakers estimated from AmbianceCount (Chen, 2020).

Consider a scenario in which $K > 0$ people are co-located and are talking in sub-groups. AmbianceCount is designed to estimate the number of concurrent speakers for each segment as belonging to one of 11 classes $\{1, 2, 3, 4, 5, 6, 7, 8, 9, 10, >10\}$. As shown in Figure S1, the system consists of a front-end utterance embedding extractor and a backend classifier.

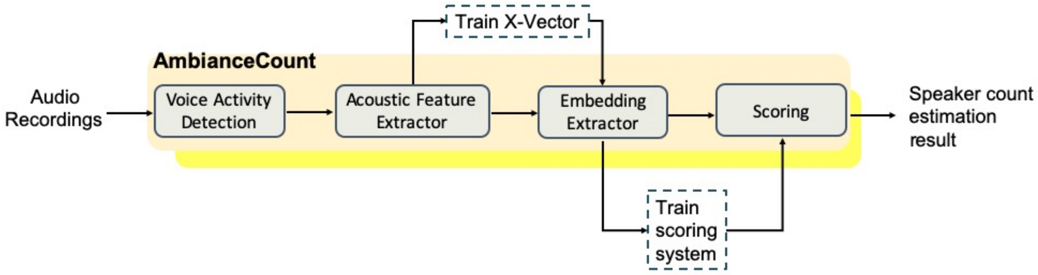

Figure S1: The overview of AmbianceCount

1.1 Acoustic Features Extraction

Filter Banks and pitch information are extracted as acoustic features and are fed as input representations to neural networks. Such representations can reduce redundancy of signals and capture significant sound characteristics, like phase information or periodicity.

1.2 Embedding Extractor

The performance of AmbianceCount highly depends on the ability of the embedding extractor to capture speech mixture characteristics. As shown in Figure S2, the embedding extractor consists of a SpecAugment module (Park et al., 2019) that augments the training data, a ResNet-based (He et al., 2016) neural network that extracts frame-level features, an attentive statistics pooling layer (Okabe et al., 2018) that turns frame-level features to embeddings, and a classification-regression objective function that helps the machine estimate speaker count in a human's perspective.

SpecAugment (Park et al., 2019) is applied to augment the training data by operating directly on the acoustic features, as if they were images. Augmented data are then fed into a ResNet34 (He et al., 2016) to extract frame-level features (25ms) which can best discriminate between different speech mixtures.

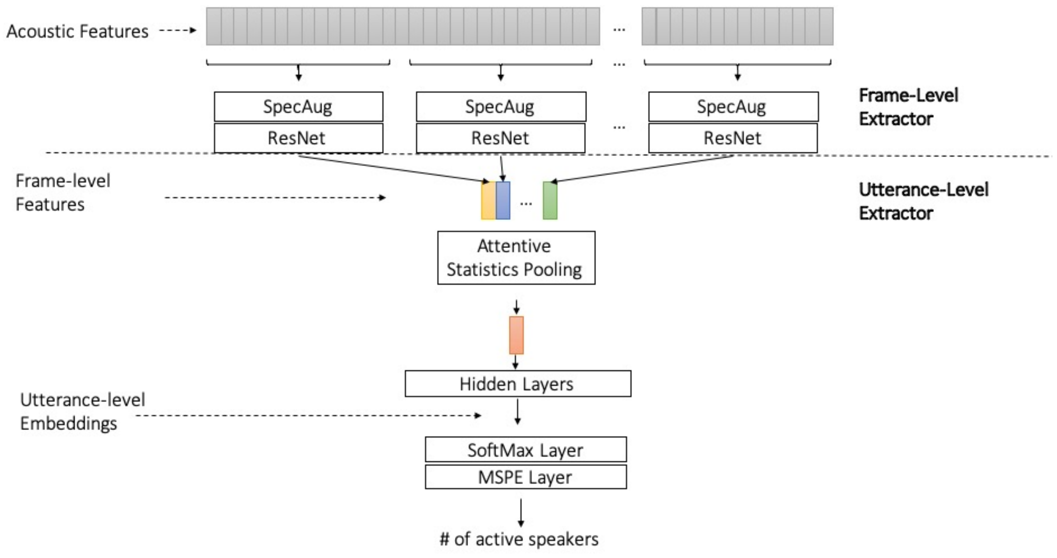

Figure S2: Architecture of embedding extractor: extracting utterance-level embeddings from speech mixtures

Frame-level features (25ms) extracted from neural networks are concatenated and processed to form an *utterance-level embedding* (5s). Instead of averaging all frames equally, the *attentive statistics pooling* proposed by (Okabe et al., 2018) is utilized to enable embeddings to focus on important frames and thus to obtain utterance-level representations with higher discriminative power.

Given that humans are in general more discriminative when there are fewer sources in the mixture and most errors fall into adjacent classes, a *classification-regression objective function* is proposed to jointly minimizes Cross-Entropy and Mean Square Percentage Error (MSPE) loss (Botchkarev, 2018). While retaining the advantages of cross-entropy, the loss function also keeps the relation information between classes by calculating the relative deviation from ground truth. This is achieved by jointly optimizing:

$$\mathsf{L}_{\text{Cross Entropy}} = -\frac{1}{n}\sum_{i=1}^{n} t_i \cdot \log(y_i) \tag{S1}$$

$$\mathsf{L}_{\text{MSPE}} = \frac{1}{n}\sum_{i=1}^{n}\left(\frac{|z_i - t_i|}{|t_i|}\right)^2 \tag{S2}$$

$$\text{Loss} = \alpha \cdot \mathsf{L}_{\text{Cross Entropy}} + \beta \cdot \mathsf{L}_{\text{MSPE}} + \frac{\lambda}{2}\|\boldsymbol{W}\|_2^2 , \tag{S3}$$

where $\alpha, \beta \in [0, 1]$ represent weights of classification and regression objectives, t_i is the ground truth, y_i and z_i the output of classification layer and regression layer respectively, $\boldsymbol{W}$ denotes all trainable network parameters, and λ is the weight decay, which is applied to avoid over-fitting.

1.3 Backend Scoring

As shown in Figure S3, the backend system consists of three blocks, Correlation Alignment (CORAL) (Sun et al., 2015), Linear Discriminant Analysis (LDA) and Interpolated Probabilistic LDA. For scoring, a similarity score between embedding pairs is computed to determine whether an embedding belongs to class i or not. The key is to find a subspace to maximize inter-class differences and minimize the intra-class differences, which is accomplished by Linear Discriminant Analysis (LDA) and Interpolated Probabilistic LDA. For domain adaptation, a recently introduced domain adaptation algorithm called CORAL (Sun et al., 2015) is leveraged to align the distributions of out-of-domain and in-domain features in an unsupervised way. Details of the architecture, model selection, training and evaluation can be found in our previous work (Chen, 2020).

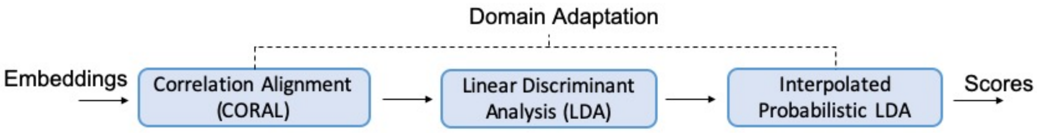

Figure S3: Overview of the backend system

2 SYNTHETIC DATA GENERATION

2.1 Synthetic speech mixture preparation

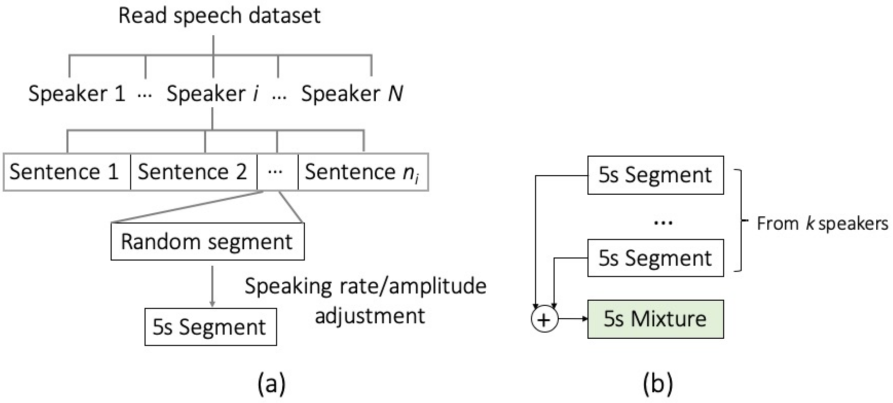

Figure S4: Synthetic data preparation: (a) generating 5s speech segments; (b) creating K-speaker mixtures.

Figure S4 describes how to create a K-speakers mixture from LibriSpeech corpus (Panayotov et al., 2015), a speech dataset based on LibriVox's audio books, with 1228 female speakers and 1210 male speakers. For each speaker, a 15-30 min recording is generated by concatenating all the sentences from that speaker. Then an audio segment is randomly selected from each recording. Next, audio segments are randomly adjusted in volume and speaking rate to simulate how people speak in real-world scenarios. Consider the influence of microphone locations as well as people speaking in different volumes and speeds, we randomly apply to the mixture a volume factor between -3 dB to +3 dB and a speed factor ranging from -0.9 dB to +0.8 dB. Finally, adjusted segments from K speakers are trimmed to T seconds and overlapped with each other to generate a speech mixture, labeled with speaker count of K. At this point, we have what

we call a clean speech mixture, in which at any given time, K speakers are simultaneously active, without noise and reverberation.

The same procedure can be applied to additional datasets like TIMIT (English) (Garofolo, 1993) and THCHS (Mandarin) (Wang and Zhang, 2015) for the evaluation process.

2.2 Creating real-world scenarios

Table S1. Sound effects for creating scenarios

Sound effects	Parameters	Sound Effect Library
Background noise	SNR:0-18 dB	MUSAN-NOISE, MUSAN-MUSIC
Foreground noise	SNR:0-18 dB	MUSAN-NOISE, MUSAN-MUSIC
Reverberation	Small/Median/Large room	Simulated room impulse responses (RIRs)

As is summarized in Table S1, thousands of realistic scenarios are simulated by combining three types of sound effects: background noises, foreground noises and reverberation.

Usually, background noises either sound weaker or are continuous enough to make up the background texture of a soundscape. So in our process, background noises are added to the entire recording, and repeated as necessary to cover the full length. On the contrary, foreground noises, standing out against the background, are added sequentially, according to a specified interval, and do not overlap. To build a diverse background or foreground noise, we combine MUSAN-NOISE and MUSAN-MUSIC (Snyder et al., 2015), and get a sound effect dataset that covers the sound of things (*e.g.*, dial tones, fax machine noises), natural sounds (*e.g.*, thunder, wind), and music without vocal(*e.g.*, Western art music and popular genres). Finally, the speech mixtures reverberate via convolution with simulated room impulse responses (RIRs) described in (Ko et al., 2017), which is to simulate how the mixtures sound like in different room settings.

The same procedure can be applied to additional datasets like TIMIT (English) (Garofolo, 1993) and THCHS (Mandarin) (Wang and Zhang, 2015) for the evaluation process.

REFERENCES

Botchkarev, A. (2018). Performance metrics (error measures) in machine learning regression, forecasting and prognostics: Properties and typology. *arXiv preprint arXiv:1809.03006*

Chen, W. (2020). Ambiancecount: An objective social ambiance measure from unconstrained day-long audio recordings

Doukhan, D., Carrive, J., Vallet, F., Larcher, A., and Meignier, S. (2018). An open-source speaker gender detection framework for monitoring gender equality , 5214–5218

Garofolo, J. S. (1993). Timit acoustic phonetic continuous speech corpus. *Linguistic Data Consortium, 1993*

He, K., Zhang, X., Ren, S., and Sun, J. (2016). Deep residual learning for image recognition. In *Proceedings of the IEEE conference on computer vision and pattern recognition*. 770–778

Ko, T., Peddinti, V., Povey, D., Seltzer, M. L., and Khudanpur, S. (2017). A study on data augmentation of reverberant speech for robust speech recognition , 5220–5224

Okabe, K., Koshinaka, T., and Shinoda, K. (2018). Attentive statistics pooling for deep speaker embedding. *arXiv preprint arXiv:1803.10963*

Panayotov, V., Chen, G., Povey, D., and Khudanpur, S. (2015). Librispeech: an asr corpus based on public domain audio books , 5206–5210

Park, D. S., Chan, W., Zhang, Y., Chiu, C.-C., Zoph, B., Cubuk, E. D., et al. (2019). Specaugment: A simple data augmentation method for automatic speech recognition. *arXiv preprint arXiv:1904.08779*

Snyder, D., Chen, G., and Povey, D. (2015). Musan: A music, speech, and noise corpus. *arXiv preprint arXiv:1510.08484*

Sun, B., Feng, J., and Saenko, K. (2015). Return of frustratingly easy domain adaptation. *arXiv preprint arXiv:1511.05547*

Wang, D. and Zhang, X. (2015). Thchs-30: A free chinese speech corpus. *arXiv preprint arXiv:1512.01882*
